# Lethal and Sublethal Effects of Pyriproxyfen on *Apis* and Non-*Apis* Bees

**DOI:** 10.3390/toxics8040104

**Published:** 2020-11-17

**Authors:** James Devillers, Hugo Devillers

**Affiliations:** 1 CTIS, 69140 Rillieux La Pape, France; 2 SPO, INRAE, Montpellier SupAgro, University of Montpellier, 34000 Montpellier, France; hugo.devillers@inrae.fr

**Keywords:** pyriproxyfen, honey bee, bumble bee, solitary bee, stingless bee

## Abstract

Pyriproxyfen is a juvenile hormone mimic used extensively worldwide to fight pests in agriculture and horticulture. It also has numerous applications as larvicide in vector control. The molecule disrupts metamorphosis and adult emergence in the target insects. The same types of adverse effects are expected on non-target insects. In this context, the objective of this study was to evaluate the existing information on the toxicity of pyriproxyfen on the honey bee (*Apis mellifera*) and non-*Apis* bees (bumble bees, solitary bees, and stingless bees). The goal was also to identify the gaps necessary to fill. Thus, whereas the acute and sublethal toxicity of pyriproxyfen against *A. mellifera* is well-documented, the information is almost lacking for the non-*Apis* bees. The direct and indirect routes of exposure of the non-*Apis* bees to pyriproxyfen also need to be identified and quantified. More generally, the impacts of pyriproxyfen on the reproductive success of the different bee species have to be evaluated as well as the potential adverse effects of its metabolites.

## 1. Introduction

Pollination is an ecosystem service freely delivered worldwide by a plethora of taxa including more than 20,000 described bee species inhabiting every continent except Antarctica as well as butterfly, fly, ant, beetle, bat, and bird species showing various levels of specialization in their pollinating activity [1,2,3]. Nearly 90% of the wild flowering plants depend, at least in part, on the transfer of pollen by animals while pollinator-dependent crops only contribute to 35% of global crop production volume [3,4,5].

*Apis mellifera* (Hymenoptera: Apidae) is the main bee species used in numerous countries for crop pollination and honey production represents a substantial source of income, particularly in rural communities [6]. Among the non-*Apis* bees, the bumble bees (Hymenoptera: Apidae) are very efficient and reliable pollinators of greenhouse crops favoring the production of fruits of quality due to their buzzing behavior, high speed of pollination, and their ability to fly and forage under cool temperatures as well as under cloudy and low light conditions [7]. The most widely used species for crop pollination are *Bombus terrestris* and *B. impatiens* [8]. The stingless bees (Hymenoptera: Apidae, Tribe Meliponini) are social bees living in the tropical and subtropical regions of the world in which they highly contribute to the pollination of numerous wild plants and cultivated species. Some stingless bee species are managed to promote pollination of diverse crops [9,10]. Thus, for example, in the Northeastern region of Brazil, *Melipona subnitida* is successfully used for the pollination of greenhouse sweet pepper (*Capsicum annuum*) [11]. In the same way, in Costa Rica, 55 insect species were found visiting and pollinating chayote flowers (*Sechium edule*, Cucurbitaceae), but the only important ones, on the basis of abundance and efficiency, were 28 species of stingless bees. Of these species, *Trigona corvina* and *Trigona* (*Partamona*) *cupira* were the most important [12]. Other successful results of crop pollination by stingless bee species have been described by Heard [9] and Slaa et al. [13].

Solitary bees have been also used for a long time for crop pollination. The longest managed species is undoubtedly *Megachile rotundata* (Hymenoptera: Megachilidae), the alfalfa leafcutting bee that was accidentally introduced into the United States in the 1940s. The bee has boosted the alfalfa seed industry by tripling its production [14]. The alkali bee, *Nomia melanderi* (Hymenoptera: Halictidae), is also an effective alfalfa pollinator [15]. The blue orchard bee, *Osmia lignaria* (Hymenoptera: Megachilidae), shows a strong preference for collecting pollen and nectar on fruit tree flowers. Management methods to use this bee to pollinate apples, cherries, and almonds have been developed in North America. The use of the hornfaced bee (*O. cornifrons*) in Japan has considerably increased the production of apples. The horned bee (*O. cornuta*) is successfully used in Europe for pollinating almond, pear and apple orchards [16,17,18]. The red mason bee (*O. bicornis*) has found applications to pollinate fruit trees but also crops such as strawberries (*Fragaria* spp.) and oil seed rape (*Brassica napus*) [19,20].

In contrast to the honey bees, stingless bees, and bumble bees that are social insects living in colonies, the solitary bees build and provision their nest and raise their offspring alone, without cooperation from other individuals. It seems that the higher the degree of sociality the better the capacity to respond to stressors [21].

Over the past decades, collapses of wild and domestic bee populations have been reported in various parts of the world [22,23,24,25,26]. Many hypotheses have been put forward to explain these phenomena but now, it is frequently admitted that no single factor can explain the decline of bee populations. The involved factors include habitat destruction and fragmentation, intensive beekeeping practices, climate change, decreased abundance and diversity of floral resources, and the action of biological agents and xenobiotics [27,28,29,30].

Among the man-made chemicals, the pesticides used in agriculture highly contribute to the decline of bees. Herbicides curtail weed spread in crops but their use reduces the availability and diversity of flowers for pollinators as well as the attractiveness of the landscapes to bees [31,32]. Insecticides directly act on bees, leading to lethal or sublethal effects depending on their mechanism of action, the dose used, the exposure route, and so on. Although the first studies on the adverse effects of insecticides to non-*Apis* bees date back to about 75 years [33,34], their ecotoxicology is less documented than that of the western honey bee (*A. mellifera*) [35]. In addition, there is a strong discrepancy between the categories of non-*Apis* bees. The studies on the toxicity of pesticides against the bumble bees are fairly abundant [36,37,38,39,40,41,42,43,44,45,46,47,48,49,50]. On the contrary, the toxicity studies are limited on stingless bees [51,52,53,54,55,56,57]. As regards solitary bees, there are great disparities between taxa. Very few studies are available on the effects of pesticides on *N. melanderi* [38,58,59], whereas the ecotoxicology of *M. rotundata* is well documented [38,40,58,59,60,61,62,63,64,65,66]. This is also the case of *Osmia* spp. [40,62,65,66,67,68,69,70,71,72,73,74].

Due to the historical use of the honey bee as pollinator in agriculture and the economic importance of beekeeping, the first publications on the effects of insecticides on *A. mellifera* date back to the end of the 19th century [75] and the increase of their number has followed the development of the industry of synthetic chemical pesticides [76].

Current regulatory risk assessment schemes for evaluating the potential adverse effects of pesticides rely on the use of the honey bee as a surrogate for all the other bee species. In addition to the acute toxicity tests, different test protocols have been developed to evaluate the sublethal and behavioral effects of pesticides against the honey bee [77,78,79,80,81].

In this context, the aim of this study was to evaluate the toxicity of pyriproxyfen on the *Apis* and non-*Apis* bees and if necessary, to clearly identify the gaps that need to be filled. Pyriproxyfen is an insect growth regulator that mimics the juvenile hormone activity. By disrupting the hormonal system of the insects, pyriproxyfen interferes with the development of the larvae and their transformation into adults [82]. It is widely used worldwide in agriculture and horticulture to fight a huge number of pest species [83,84,85,86]. In addition, among the molecules used for controlling mosquitoes [87,88,89], pyriproxyfen has shown its high efficacy as larvicide against all the mosquito species [90,91,92,93,94,95]. As a result, this insecticide is widely spread in the environment. Its fate in the aquatic and terrestrial ecosystems has been recently reviewed [96,97], as well as its aquatic ecotoxicity [96]. Consequently, there is a need to evaluate the adverse effects of pyriproxyfen on the *Apis* and non-*Apis* bees due to the pivotal importance of bees in the terrestrial ecosystems. Because the first registrations of pyriproxyfen for pest control date back to the beginning of 1990s [98], the present review represents the analysis of 30 years of publications on the toxicity of this insecticide to bees.

## 2. Effects of Pyriproxyfen on *Apis* Bees

### 2.1. Mortality

Pyriproxyfen is considered to have a low acute toxicity against the adult honey bees because the 48-h LD_50_ (lethal dose 50%) values via oral and contact contaminations are both greater than 100 µg a.i. (active ingredient)/bee [99,100,101]. Machado Baptista et al. [102] showed that the direct spraying of pyriproxyfen (Cordial 100^®^, EC-0.075) on Africanized workers of *A. mellifera* led to a LT_50_ (lethal time 50%) value of 466 h. Using a sprayer at 0.58 mL/s and an average spraying rate of 0.00583 mL/cm^2^ for simulating a normal field spraying, Costa et al. [103] sprayed a Tiger^®^ commercial formulation of pyriproxyfen at 0.1 g a.i./L on groups of 10 honey bees. A LT_50_ >100 h was found. At the end of the 72 h test period, the mortality was of 30.2%. Diet consisting of 20 mL of honey and 50 g of sugar was contaminated in surface (7.06 cm^2^) with Tiger^®^. A LT_50_ value of 166.83 h was calculated. A mortality of 16.7% was recorded and no behavioral disorders were observed at the end of the experiment. Interestingly, a contact toxicity test was also performed with contaminated melon (*Cucumis melo*) leaves by surface spraying with Tiger^®^ (0.00583 mL/cm^2^). After drying, the leaves were placed in the test container with diet and water before the introduction of the adult bees. After 72 h, only a mortality of 34.7% was observed and a LT_50_ value of 100.58 h was calculated. A rather similar investigation was performed by Naiara Gomes et al. [104]. Field concentration of Tiger^®^ recommended for the control of the whitefly (*Bemisia tabaci*) and the 1/10 dilution (i.e., 62.50 and 6.25 µL/L) were tested. Survival via ingestion was conducted on foragers in Petri dishes. No significant mortality was recorded after 24 h in comparison with the controls. It is noteworthy that the surviving bees were used to perform a flight bioassay in a metal frame flight tower (H = 105 cm) with only a light source at the top. Both concentrations of pyriproxyfen induced a change in the flight behavior of the bees because most of them remained at the bottom of the tower. A residue contact test on the leaves of “Rock” (hybrid) cantaloupe melon plants with the recommended field concentration was performed according to Costa et al. [103]. Although after one hour a mortality of about 35% was recorded, the percentage of mortality of the adult workers after 24 and 48 h did not significantly differ from the control. Lastly, it is interesting to note that Naiara Gomes et al. [104] also tested the repellency potential of pyriproxyfen by using a test tube with contaminated food at the field concentration. At the end of the test, fewer than 10% of the bees avoided the pyriproxyfen-contaminated diet, revealing no repellency effect. This lack of repellency may imply that the bees are not able to avoid pollens and nectars contaminated by pyriproxyfen residues.

Cages with 30 to 40 foragers were set in a wind tunnel built to simulate the droplet size of the aerial applications of pesticides in fields [105]. The cages were exposed to an atomized liquid treatment of NyGuard^®^ (10% pyriproxyfen) at 0.5, 1, 2, and 3 times the label dose corresponding to 0.53, 1.06, 2.12, and 3.18 mL/L H_2_O. A control without insecticide was also used. After treatment, the cages were put in plastic holding units including feeders with a 50:50 sucrose solution and placed in an incubator at 34 °C. Mortality was checked every 24 h for 10 consecutive days. Fisher et al. [105] showed that only the application doses of pyriproxyfen above the label dose significantly affected the average forager mortality after 10 days compared with the untreated control group.

Interestingly, Phillips [106] showed that the absorption of pyriproxyfen and consequently its toxicity highly depended on the solvent used. Thus, for example, acetone might cause an underestimation of the adverse effects due to a restricted absorption. On contrary, DMSO (dimethyl sulfoxide) significantly enhances the absorption of pyriproxyfen.

The oral and contact LD_50_ values (µg/bee) are commonly used with the recommended field treatment doses (g/ha) for calculating the hazard quotients (HQs). Thus, for example, with the highest field application rate of 225 g a.i./ha selected by EFSA [101] for pyriproxyfen and a contact or oral LD_50_ on *A. mellifera* >100 µg a.i./bee, a HQ <2.3 (i.e., 225/100) is obtained. Poquet et al. [107] have tried to refine the HQ calculation when the pesticides, including pyriproxyfen, are sprayed. In the same way, Cunha et al. [108] included drift deposition in the equation to account for the risk outside the applied area of citrus cultures in which pyriproxyfen was sprayed.

### 2.2. Effects on Development

Africanized *A. mellifera* bees contaminated with pyriproxyfen during their development showed alterations in the pigmentation and sclerotization of their cuticle [109]. Fifth-instar larvae at the feeding phase (LF5, 4–4.5 days after hatching (d.a.h.)) were treated topically in their comb cells with pyriproxyfen (1 μg in 1 μL of acetone). Controls were left untreated or only received 1 μL of acetone. After treatment, the combs were put back in place within the hives. At the beginning of the pupal period (about 8.5–9 d.a.h.), pupae were removed from the combs and introduced in an incubator at 34 °C and with 80% of relative humidity (RH) until their emergence. Fifth-instar larvae at the spinning phase (LS5, 5–6 d.a.h.), prepupae (PP, 7–8 d.a.h.), white-eyed, unpigmented cuticle pupae (Pw, 8.5–9 d.a.h.), pink-eyed, unpigmented cuticle pupae (Pp, 10 d.a.h.), dark-pink-eyed, unpigmented cuticle pupae (Pdp, 11 d.a.h.), and brown-eyed, unpigmented cuticle pupae (Pb, 12–13 d.a.h.) were also contaminated with 1 μg of pyriproxyfen. Topical application of pyriproxyfen to LS5 or PP or even to Pw led to an anomalous pigmentation in the pupae. Thorax and head were precociously and strongly pigmented. Treatments from Pp to Pb did not impact the cuticular pigmentation process. Eye pigmentation did not appear in pupae treated at LF5. When applied from LS5 to Pw, the anterior edges of the eyes did not darken. Brown-eyed medium pigmented cuticle pupae (Pbm, 14–15 d.a.h.) treated with pyriproxyfen at the Pw phase showed a higher phenoloxidase activity, an important enzyme in melanin biosynthesis, than the controls and the effect was dose-dependent. Esterase-6 activity was also detected earlier than in the controls [109]. Bitondi et al. [109] also showed that LF5 contaminations blocked the pupal development and the immatures died. LS5, PP, and Pw treatments fastened the pupal development but emergence was impaired. Pp, Pdp, or Pb contaminated pupae emerged before the controls.

These results were completed by those of Zufelato et al. [110] who showed that 1 μg of 20-hydroxyecdysone (20E) injected to Pw did not affect the beginning of pigmentation or its intensity. On the contrary, at Pp, the pigmentation process was impacted. Injection of 2 or 5 μg of 20E at Pw delayed pigmentation. Treatment at the Pb stage was very detrimental. Topical application of 1 μg of pyriproxyfen at Pw led to a delay of the pupal ecdysteroid peak by about 4 days.

Santos et al. [111] demonstrated that the topical application of 1 μg of pyriproxyfen in acetone to Pw bees accelerated the transition phase of epidermal protein pattern. The protein pattern at 96 h post-treatment was similar to that observed much later in the controls (i.e., 192 h). On the contrary, 20E injected to Pdp or Pb bees led to an arrest in the protein pattern development. A double hormonal treatment restored the normal temporal expression of epidermal proteins. In the same way, Boleli et al. [112] showed that 1 μg of pyriproxyfen in 1 μL of acetone topically applied to the dorsum of prepupal abdomen of Africanized *A. mellifera* resulted in eyes with an unpigmented anterior region. At 5 μg of pyriproxyfen the entire eye was unpigmented. Unorganized retinula and apoptic figures were observed.

Pinto et al. [113] showed that pyriproxyfen influenced the synthesis, secretion, and accumulation of vitellogenin in Africanized *A. mellifera*. Groups of 120 newly emerged worker bees were contaminated by topical application of 10, 5, 2.5, 1.25, 0.1, 0.01, or 0.001 μg of pyriproxyfen in 1 μL of acetone. A control group only treated with 1 μL of acetone was also included. Bees were placed into small cages in an incubator for six days. Pollen and water were given in enough quantity to secure vitellogenin synthesis. In the controls, vitellogenin titer, measured by immunoelectrophoresis, increased from day four to day six of adult life. Pyriproxyfen inhibited this effect when topically applied at 10 μg to newly emerged workers. Consequently, the very low vitellogenin concentrations found in the 4–6-day-old treated bees were similar to those measured in 0–3-day-old bees. The lack of vitellogenin accumulation in treated bees was shown via the hemolymph total protein content. At day 6, the 10 μg treated bees showed significantly lower protein titer than the controls of the same age. In the 6-day-old bees, the dose-dependent inhibitory effect of pyriproxyfen was obvious. After treatment with 1.25 to 10 μg of pyriproxyfen, protein titers declined progressively. The normal accumulation of protein in hemolymph did not change with 0.1, 0.01, or 0.001 μg of pyriproxyfen. The low vitellogenin titer in the hemolymph of the contaminated bees was not the result of its increased sequestration by ovaries. Treated bees did not have vitellogenic ovaries. Pinto et al. [113] also showed that fat bodies of bees treated with 10 μg of pyriproxyfen synthesized and secreted less vitellogenin in vitro than the controls.

Because flight musculature is crucial for the foraging and mating activities of the honey bee, Corrêa Fernandez et al. [114] investigated the effects of pyriproxyfen (1, 100, and 1000 ng/µL) on the flight muscle differentiation in Africanized *A. mellifera* by using the chronic larval test developed by Aupinel et al. [115,116,117]. Independent of the tested concentration, the differentiation of the flight musculature was delayed in comparison to the control.

The hypopharyngeal glands that are responsible for the royal jelly secretion by the nurses show a flexible secretory activity depending on the needs for feeding brood [118]. Their development can be assessed by measuring the total protein contents of the glands after their extraction by using the Bradford method [119]. It is worth noting that Heylen et al. [120] showed that fenoxycarb, which is also a juvenile hormone mimic, adversely affected the hypopharyngeal glands. These authors indicated that fenoxycarb “acted as an inducer of precocious foraging”. In this context, the effects of pyriproxyfen on the development of hypopharyngeal glands were assessed by Devillers et al. [121] by using the chronic larval test of Aupinel et al. [115,116,117]. Each larva received a cumulative dose of 54 ng of pyriproxyfen (98%) via diet. The emerging bees were reared in the laboratory until the age of 10 days. During this period, they were fed ad libitum with pollen and syrup. At day 26, the bees were killed and the protein contents of the hypopharyngeal glands were measured by the Bradford method. A significant reduction in the protein contents was observed. It was deducted that the sublethal dose of 54 ng/larva highly perturbed the activity of the nurses within the hive.

### 2.3. Lethal, Sublethal, and Behavioral Effects at the Hive Level

Some attempts have been made to evaluate the effect of pyriproxyfen at the hive level either from in situ studies or by using apparatus allowing to better integrate the potential effects of the insecticide on the whole colony.

Thus, Utsumi et al. [122] at Sumitomo Chemical Co. Ltd. (Japan) tested the potential adverse effects of pyriproxyfen (10% EC) on *A. mellifera* colonies under field conditions. Four colonies including 60,000 to 80,000 workers were used per group. Each group was placed within 0.5 ha located at 3 km from full-flowering *Phacelia tanacetifolia*. A treatment at 75 g a.i./ha was applied. Both the eggs and larvae in the combs developed into healthy adult bees and no differences were found with the controls. Continuous trap monitoring showed that the number of dead bees after treatment was lower than that observed during the hive acclimation period and was the same as for the untreated colonies. There was no difference between the treated and untreated groups as regards the hive weight during the two months of observation after the experimental contamination by pyriproxyfen. However, it is worth noting that the study has an insufficient sample size (i.e., four colonies per treatment group) to be able to see a significant difference in colony weight due to pyriproxyfen exposure if a difference existed.

Four colonies placed in hives of nine frames were used by Chen et al. [123] and Ko et al. [124] for estimating the impact of pyriproxyfen (11% EC) on the larval development. Fifty brood cells containing one-day-old larvae were randomly selected and marked for each treatment that consisted in the total ingestion of 0, 4, 40, 400, and 4000 pg of pyriproxyfen per larva in their diet (0, 0.1, 1, 10, and 100 mg/kg). After the treatment, the frames were returned to the hives. Mortality and capping rates were recorded at day 7. At day 13, the pupae in the marked areas were taken out from the capped cells, put into plates and placed in an incubator (34 °C, 70% RH) until emergence. A high mortality was observed at the two highest doses leading to capping rates of 22.2% and 0%, respectively. The results were statistically significant. As regards the two other doses, the capping rates, days to emergence, and the hatching rates were not significantly different from the control with the basic larval diet and the unfed control. However, it is worth noting that at 1 mg/kg the percentage of deformed wings was statistically significant.

In another experiment, 12 colonies were used. Each hive was divided with a queen excluder in part A (four frames) and part B (five frames). The queen was first put in part B for three days. This resulted in the absence of eggs in part A. Then, the queen was moved to part A to lay during 24 h and 100 eggs were marked on the transparent slide covering the frame. After three days (day 4) the queen was transferred again to part B for one day and then 100 eggs were marked. Queen exchange was made at day 1, 4, 7, 10, 13, 16, 19, 22, and 25 leading to the identification of the groups 1 to 9. At day 13, the colonies were contaminated with a syrup including 10 or 100 mg/kg of pyriproxyfen. The control group was only fed with syrup (50%). Hatching and capping rates were recorded at day 5 and 11, respectively. At day 17, the pupae were extracted and placed in plates that were kept in an incubator until the emergence of the bees. At both doses, numerous pupae died showing a black cuticle or failed to emerge. Egg hatching rate decreased at both doses. Larval capping rates followed a similar pattern. Hatching rate was significantly more reduced at 100 mg/kg than at 10 mg/kg. At both treatments, newly emerged adults showed deformed wings. There was an important decrease in the weight of royal jelly per cell at 100 mg/kg [123,124].

Fourrier et al. [125] used the test of Aupinel et al. [115,116,117] to chronically contaminate larvae of honey bees with actual cumulative doses of 23 ng and 57 ng of pyriproxyfen (98%) per larva. Water and acetone (99%) controls were included. Emerging bees were collected between day 18 and day 20. They were tagged and then placed into an incubator (35 °C, 50% RH) for 24 h before their introduction in a colony. Malformed emerging bees were recorded but they were excluded from the experiments. Tagged bees of one day old were reintroduced from day 19 to day 21 in a four-frame observation hive that included combs for brood, honey, pollen, and storage with about 6000 bees and a fertile queen of one year old. Food was supplied via a feeder. The hive was placed into an outdoor flying cage with a plastic sheet at the bottom for collecting the dead bees. After bee reintroductions, the nestmate aggressive behavior consisting in biting and pushing was recorded daily from 9:00 to 11:00 a.m. for five days. Social and non-social tasks were recorded twice a day (i.e., 11:00 to 12:30 a.m., 2:00 to 4:00 p.m.) for 15 days starting three days after the first bee introductions.

During the assay, pyriproxyfen did not lead to an increase in the mortality but induced a slightly shorter development time than in the controls. Both doses induced malformations in bees (damaged and atrophied wings). The rejection rate was of 38.10% at 23 ng of pyriproxyfen and 74.65% at 57 ng versus 6.38% for the control water and 8.05% for the control with acetone. All the above results were statistically significant. Bitondi et al. [109] also noted that the larvae contaminated with pyriproxyfen and then reintroduced into the colonies were subject to exclusion. However, this interesting observation was not quantified.

Fourrier et al. [125] tried to gain insights into the mechanism leading to the frequent rejection rate of the young contaminated bees by nestmates that undoubtedly leads to their death. They found differences between the cuticular hydrocarbon profiles of the control and treated bees. It is very interesting because the cuticular hydrocarbon profiles play a key role in nestmate recognition [126]. Fourrier et al. [125] also demonstrated that exposure to 23 and 57 ng of pyriproxyfen changed the social behavior of the bees. Young exposed bees performed more non-social tasks (self-grooming, inactivity, walking) than the non-contaminated bees used as control. In addition, the behavioral effects were dose-dependent [125].

Foragers commonly gather nectar and pollen within 1.5 km of their hive but exceptionally the foraging flights can be of about 10 km, depending on the need for food and its availability [35]. Foragers also visit puddles, ponds, and other aquatic media for collecting the 10 to 40 L of water that are necessary yearly for the colony depending on its size [127]. As a result, the hive products and bees can be contaminated by the pyriproxyfen used in agriculture and in vector control.

Thus, among 836 samples of wax, pollen, and bees analyzed by Mullin et al. [128], pyriproxyfen was found twice in wax at the concentrations of 2.2 µg/kg and 7.6 µg/kg (LOD = 1 µg/kg). Calatayud–Vernich et al. [129] measured residues of pyriproxyfen at the concentration of 25 µg/kg in capping bee wax (LOD = 0.7 µg/kg, LOQ = 2 µg/kg). Wiest et al. [130] found pyriproxyfen in 4% of the honey samples (LOD = 1.5 µg/kg, LOQ = 4.3 µg/kg), in 1% of the honey bees (LOD = 2.1 µg/kg, LOQ = 4.3 µg/kg) and in 5% of the pollen samples (LOD = 2.1 µg/kg, LOQ = 8.6 µg/kg) analyzed. All the concentrations found were inferior to the LOQs. In another study, these authors found pyriproxyfen in 1.4% of the honey bee samples (*n* = 141), 3.5% of the honey samples (*n* = 141), and 4.7% of the pollen samples (*n* = 128). Again, the concentrations found were inferior to the LOQs [131]. Vidau [132] detected pyriproxyfen in 11.1% of the pollen samples analyzed (LOQ = 10 µg/kg) while Calatayud–Vernich et al. [133] found pyriproxyfen in one of the 45 pollen samples analyzed at a concentration of 6 µg/kg and in 12% of the beebread samples analyzed (*n* = 33) at concentrations ranging from 1 to 5 µg/kg [134]. During a two-year study performed in IPM (integrated pest management) citrus orchards, Garcia–Valcárcel et al. [135] found pyriproxyfen at concentrations up to 43.2 µg/kg in fresh pollen samples and up to 3.4 µg/kg in honey bees. Lastly, among 155 fresh pollen samples collected from September 2012 to August 2013 in 14 monitoring apiaries in Taiwan, Nai et al. [136] found pyriproxyfen only once at a concentration of 40 µg/kg (LOD = 10 µg/kg).

A survey performed in Italy from 2015 to 2019 revealed that among the 696 samples of dead honey bees analyzed, 0.8% included pyriproxyfen with concentrations ranging from 3.7 to 5.9 ng/bee [137]. On the contrary, among 17 samples of dead honey bees analyzed by Calatayud–Vernich et al. [134], two (11.8%) included residues of pyriproxyfen at concentrations of 4 µg/kg and 558 µg/kg.

It is noteworthy that although pyriproxyfen presents a low water solubility value (0.367 mg/L at 25 °C) and a high 1-octanol/water partition coefficient (log Kow = 5.37) [96], its detection is low in the hive products and bees. However, without information on the sampling and storage protocols for the samples, it is difficult to explain these results.

A large collection of sublethal effects have been measured on the larvae and the adult bees but it is not obvious to translate these individual-level effects often measured under specific conditions within the laboratory into population-level effects that are more ecologically relevant. Modeling can be helpful to reach this goal. Among the different available paradigms, the agent-based models are the most interesting. Indeed, these bottom-up stochastic computational devices allow us to simulate spatio-temporal actions and interactions of real-word entities, called agents, in order to extract their combined effects on the system as a whole [121,138,139,140,141]. In this context, an agent-based model called SimBeePop (version 1.04) was designed for predicting the various effects of the organic molecules on all the hive inhabitants (i.e., agents). The model runs from 113 parameters that define the structure and the properties of the agents and the environment. In addition, 27 parameters allow us to characterize the type, intensity, and duration of the perturbations that can be applied to individuals (e.g., nurses), groups (e.g., larval stages) or to the whole population. These perturbations can be applied punctually (e.g., one day), during a given period (e.g., weeks) or can be made during the whole simulation time. The number and characteristics of the perturbations depend on the scenario that is simulated. The characteristics of SimBeePop are described in-depth by Devillers et al. [121]. Briefly, without the application of perturbations, the model simulates the normal population dynamics of a complete honey bee colony in a Dadant hive during a two-year period or more. To our knowledge, SimBeePop is the sole model accounting for the summer and winter bee populations with all their own characteristics as well as the main factors influencing the egg-laying rate of the queen during her life. All the different categories of bees are accounted for including the eggs and the successive larval stages. In the same way, all the important events such as the polyethism and cannibalism are encoded. As a result, SimBeePop is particularly suited for modeling the multifaceted effects of endocrine disrupting chemicals. The stochasticity accounted for by the model requires repeating the simulations with the same set of parameters (e.g., *n* = 50). The outputs are graphically displayed as collections of curves (i.e., one per simulation and the mean of all simulations) differently colored for the key bee categories.

**Figure 1:**
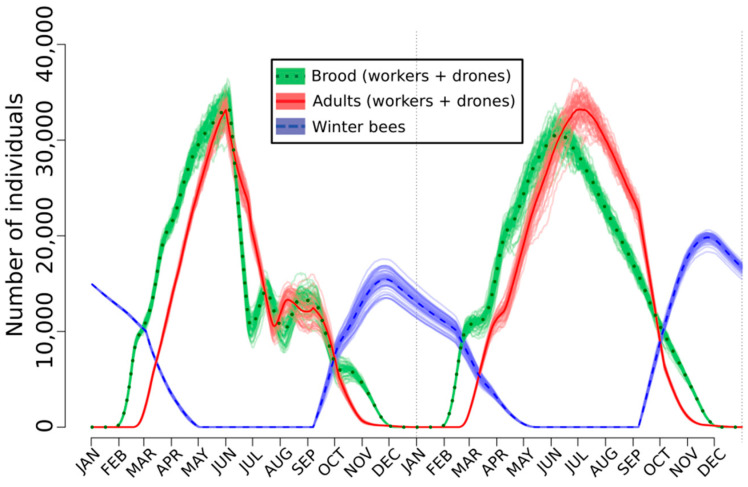
Effect of the rejection of the emergent bees on the population dynamics of *A. mellifera* simulated with the agent-based model SimBeePop.

**Figure 2:**
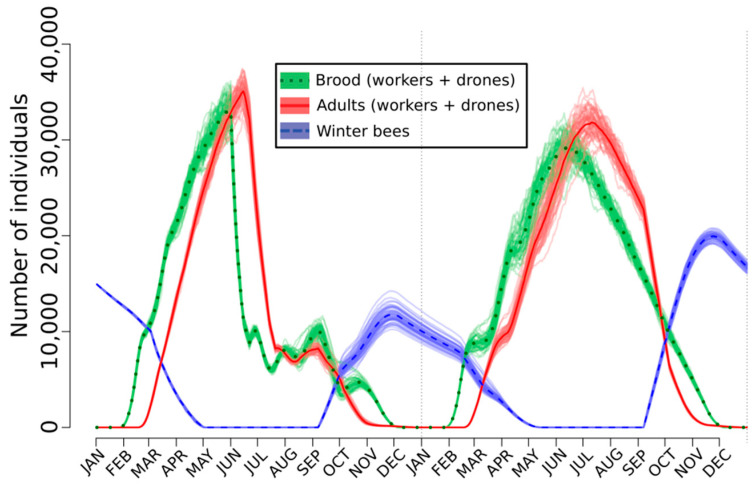
Effect of a decrease in the efficacy of the nurses on the population dynamics of *A. mellifera* simulated with the agent-based model SimBeePop.

Fourrier et al. [125] observed a rate of rejection of the emergent bees ranging from about 38% to 75%. SimBeePop allows us to test the long-term effects on the bee populations of this non-acceptance of young bees by the nestmates. To do so, a rejection rate of 50% was applied from 01 June during two months but with a linear decrease. The dynamics of 50 bee populations was simulated with the model (Figure 1). Inspection of this figure shows that after being perturbed, the normal functioning of the population was restored the second year. In Figure 2, the general simulation parameters were the same but the targets were the nurses, for which their efficacy was divided by ten. Because pyriproxyfen alters the hypopharyngeal glands, such an event is realistic and interesting to simulate. Again, the population dynamics are impacted but not enough to be affected the second year (Figure 2).

**Figure 3:**
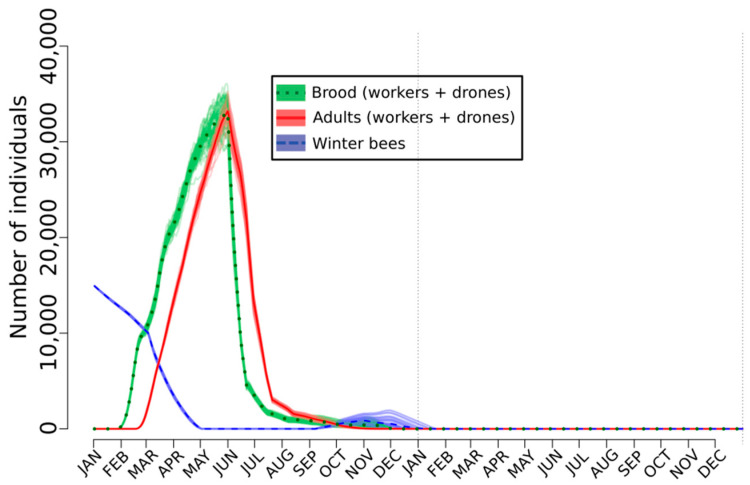
Combined effects of the rejection of the emergent bees and a decrease in the efficacy of the nurses on the population dynamics of *A. mellifera* simulated with the agent-based model SimBeePop.

When the two perturbations are simultaneously applied, the populations do not survive (Figure 3). It is a great advantage of the agent-based models for testing any kind of effects alone or simultaneously. The resilience the SimBeePop model demonstrated in Figure 1 and Figure 2 is one of the primary advantages of a social species; there are enough individuals to overcome the loss of efficiency of a few. Other scenarios have been simulated by Devillers et al. [121]. It is noteworthy that exposure scenarios to fenoxycarb have been also simulated with SimBeePop [141].

## 3. Effects of Pyriproxyfen on Non-*Apis* Bees

The non-*Apis* bees are overlooked in official risk and hazard assessment reports on pyriproxyfen. Only the recent EFSA report [101] provides information on *Bombus terrestris* and solitary bees. Thus, the acute toxicity of pyriproxyfen against *B. terrestris* by oral and contact contamination is given >72.8 µg a.i./bee and >100 µg a.i./bee, respectively. Using a field application rate of 225 g a.i./ha for pyriproxyfen, a HQ by contact less than 2.3 is proposed. As regards the solitary bees, the names of the species are not given and all the toxicity data are extrapolated from those obtained on the honey bees [101]. Inspection of the literature shows the same trends with only a little bit more toxicity results on bumble bees than other non-*Apis* bees.

De Wael et al. [142] reported on the effects of pyriproxyfen (Admiral^®^) on colonies of young *B. terrestris* (one colony per treatment group – sucrose solution with pyriproxyfen at 0, 0.2, 2, and 20 mg a.i./L). No effect on bumble bee brood development was detected. It is noteworthy that the contamination time was short (i.e., 24 h) and there was no repetition. In addition, the control was made on the smallest colony and the selected toxicity criterion lacked precision.

Adult *B. terrestris* workers were exposed by topical application and orally by drinking water and by eating pollen contaminated with pyriproxyfen (Admiral^®^ 10% EC) [143]. For each exposure route, four nests of five workers were followed for 11 weeks. Once a week, acute toxicity was evaluated. The amount of brood, brood care, egg hatching, larval mortality and number of males were recorded weekly for estimating the effect of pyriproxyfen on reproduction and larval growth. An aqueous solution at 25 mg a.i./L of pyriproxyfen was prepared for the contact and oral toxicity tests. This corresponded to the maximum field recommended concentration for Admiral^®^. For contact toxicity, 50 µL of the contaminated solution was topically applied on the thorax of each worker. Pollen was sprayed until saturation with the contaminated solution of pyriproxyfen and then given ad libitum to the nests. For each experiment, controls were made. No acute effects were observed on workers or on the number of males produced by each colony. However, a significant larval mortality was observed in the colonies treated with contaminated pollen. Adults removed dead larvae from the nests. Very often, they were third and fourth instars. In a related experiment, Mommaerts et al. [143] applied 1 µL of acetone including ^14^C-pyriproxyfen (15 mCi/g) to the thorax of adult workers. Treated individuals were sacrificed after 24 h and only 34 ± 3% of the insecticide had penetrated their cuticle. This might explain the low toxicity found for topical pyriproxyfen exposure.

To the best of our knowledge, there are no studies dealing with the acute toxicity of pyriproxyfen on stingless bees. Nevertheless, in a study on the effect of pyriproxyfen on caste development, Pinto et al. [144] showed that application of 1 µg of pyriproxyfen to spinning-stage larvae of *Melipona quadrifasciata* shifted the queen/worker ratio in developing brood to over 80% queens. Mortality rate during the pupal development was not affected by the pyriproxyfen treatment.

There is also a lack of information as regards the toxicity of pyriproxyfen to the solitary bees. In the frame of a comparison study aimed at ranking the potential toxicity of pesticides against pollinators, Mayer and Johansen [145] indicated that it was possible to apply pyriproxyfen (knack) at any time with reasonable safety to the alfalfa leafcutting bee (*Megachile rotundata*) and the alkali bee (*Nomia melanderi*).

## 4. Discussion

The information imbalance between *Apis* and non-*Apis* bees on the toxicity of pyriproxyfen is representative of what is encountered with all the other pesticides with only few exceptions such as the neonicotinoids for which the toxicity has been evaluated on different non-*Apis* bee species [36,37,40,41,43,45,46,48,49,51,57,62,66,70,71,72,74].

The situation is not surprising because the current regulations request the use of the honey bee, *Apis mellifera*, as a surrogate for characterizing the potential adverse effects of pesticides against all the bees. It is assumed that the approach is sufficiently conservative to be protective of all the other bee species, whatever their life history traits and ecological importance in the ecosystems. Our reliance on honey bees is a result of their historical uses in agriculture and beekeeping, our greater knowledge of their biology and because they are easy to rear and their high prolificacy allows us to have a great number of genetically homogeneous larvae and adults to perform the toxicity tests. Although the honey bees are undoubtedly very convenient for evaluating the toxicity of pesticides and biocides, non-*Apis* bees present ecological, physiological, and behavioral characteristics that exclude to do not consider them in the hazard and risk assessment of chemicals used in agriculture and in vector control. Recently, an invaluable comparison was made between the life history traits of the *Apis* and non-*Apis* bees and their implication for the risk assessment of pesticides [146,147,148,149]. The differences in the life history traits included the level of sociality, fecundity, nesting substrate and materials, foraging behavior, flower preference, adult food, and larval feeding behavior. The authors identified gaps in the knowledge of non-*Apis* bee biology and pesticide exposure routes that needed to be filled and that were not covered by the current *A. mellifera* exposure assessment paradigm. Thus, for example, more information is needed on the nectar and pollen consumption by the solitary and stingless bees to better quantify their dietary exposure. As regards the bees in contact with soil during a part of their life cycle, the quantification of the level of direct and indirect insecticide exposure via this medium is needed. The influence of the abiotic factors on soil exposure is also totally unknown. Because there is no need to reinvent the wheel, we only focus on specific points in relation with the environmental fate and ecotoxicity of pyriproxyfen.

Due to its mode of action, pyriproxyfen targets the larvae and their development rather than the adults. Thus, the problem to address in priority is to evaluate whether the exposure of the larvae to pyriproxyfen is equivalent for the *Apis* and non-*Apis* bees.

The main formulations of pyriproxyfen are applied to crops by spraying. When sprayed to plants, pyriproxyfen behaves as a translaminar insecticide. This means that after absorption of the insecticide by the upper surface of the leaves, a reservoir is formed within the leaf tissues allowing a residual activity as well as a potential exposure via the lower surface of the leaves [97]. Females of *Megachile* bees cut leaves of different forms for the protection of their nest and the construction of the brood cells [150]. Stingless bees can also use leaves for their nest [151]. Thus, while the larvae of honey bees and bumble bees cannot be exposed by contact with contaminated leaves, larvae of solitary bees and stingless bees are likely to be exposed to pyriproxyfen residues found in leaf tissues and on both surfaces of the leaves. It is interesting to note that in French Guiana, Remillet [152] reported that *Trigona hyalinata* and *T. pallida* are considered true pests for the flowers, young fruits and leaves of *Citrus* spp. on which pyriproxyfen is commonly used.

Due to its low water solubility and high log Kow, pyriproxyfen is adsorbed onto the soil surfaces and it is not a leacher [97]. Spray drift contributes to soil contamination by pyriproxyfen [96,97,108]. Soil is not a route of exposure for the larvae of honey bees and bumble bees but it is very relevant for solitary bees and stingless bees that nest underground. Obviously, among the above bee categories there are differences between the species.

The different uses of pyriproxyfen in agriculture and as a larvicide to control mosquitoes lead to the contamination of water media. Because a hive needs large amounts of water yearly [127], the larval exposure to contaminated water cannot be excluded. The non-*Apis* bee larvae seem to be less frequently exposed to contaminated water than the larvae of *Apis*-bees, even if among the non-*Apis* bees, those nesting underground are more exposed. More work is needed to evaluate the role of water in the contamination of bees by pyriproxyfen.

Dietary exposure via the consumption of pollen and nectar is undoubtedly an important route of exposure for the bee larvae. Pyriproxyfen has been found in various pollen samples up to about 40 µg/kg [130,131,132,133,135,136]. The number of studies on pesticide residues in nectar samples is rather limited and to the best of our knowledge, pyriproxyfen was never analyzed. Although it has been often reported that the residue levels in nectar are lower than the respective levels in pollen [153,154], there is a need to investigate the concentrations in pyriproxyfen found in nectar samples due to the mode of action and use of pyriproxyfen, as well as the variety of plant species on which it is applied [97]. Whereas the diet of the honey bee larvae is well known, there remain gaps in those of non-*Apis* bee larvae. Nevertheless, there exist differences in the diet exposure of bee larvae. The larvae of honey bees and bumble bees are continuously fed during their development (i.e., progressive provisioning), whereas the larvae of solitary bees and stingless bees are fed via a mass provision (i.e., mass provisioning) of pollen and nectar constructed by the female before laying [155,156]. In the latter case, exposure is longer than in the former situation and it is not only made by the oral route but also by contact. More generally, the larvae of bee species do not have the same size and the duration of their development can be very different. These differences influence the larval toxicity of pyriproxyfen. Consequently, although there is a convenient larval test on the honey bee [115,116,117], toxicity results on larvae of *A. mellifera* for pyriproxyfen cannot be extrapolated to the larvae of non-*Apis* bees. In this context, it is interesting to note that there are initiatives for developing larval tests for non-*Apis* bees [157,158].

Based on the multivariate analysis of toxicity results for more than 150 pesticides, Devillers et al. [38] showed that pesticides highly toxic to adults of *A. mellifera* were also highly toxic to *M. rotundata*, *N. melanderi* and *Bombus* spp. Although some exceptions were found, it is reasonable to assume that in that case, the honey bee can be used as an appropriate surrogate for estimating the short-term effects of pesticides against non-*Apis* bees. The same trend existed for pesticides that were weakly toxic against *A. mellifera* with few exceptions. Pesticides moderately toxic against *A. mellifera* showed various behaviors against the non-*Apis* bees. For these two last categories, Devillers et al. [38] warned against extrapolating results obtained on *Apis* bees to non-*Apis* bees. The differences in the intrinsic toxicity of pesticides among bee species are due to their morphology (size) but above all, their physiology [38]. Indeed, the existence of specific detoxification pathways is critically important in determining the differences in the sensitivity of the *Apis* and non-*Apis* bees to pesticides [159,160,161,162].

Based on the existing experimental results and the doses used in agriculture and in vector control, pyriproxyfen does not present an acute toxicity against the foragers. However, this does not exclude that the molecule could have adverse effects on the reproductive males and females. Indeed, there is increasing evidence that pesticides can negatively impact reproduction of *Apis* and non-*Apis* bees including egg laying, sperm viability and production [47,71,163,164,165,166]. Interestingly, Thompson et al. [163] showed that the number of queens of *A. mellifera* that successfully mated and laid eggs was affected by fenoxycarb. In the same way, Milchreit et al. [165] observed that in fenoxycarb treated colonies of *A. mellifera*, the queens suddenly stopped laying any eggs. Half of the colonies lost their queens and the remaining showed absconding behavior.

Whereas pyriproxyfen is structurally related to fenoxycarb and it impairs the reproduction process of numerous aquatic organisms [96], to the best of our knowledge, the potential effects of the molecule on the reproductive success of the different bee species have never been investigated. Because of the differences in the bee colony structures and their development cycles, extrapolation from results obtained only on *A. mellifera* would be highly questionable. Thus, for example, it is well known that under natural conditions, to hibernate, bumble bee queens dig into well-drained soil cavities and stay for several months depending on the species and the spring temperatures. In addition, following their emergence, hibernated queens are commonly found foraging on adjacent plants [167]. As a result, we claim that the risk of contamination to a honey bee queen is different from the one of a bumble bee queen and toxicity extrapolations between species, especially for chemicals acting as endocrine disruptors, would be dangerous.

Degradation of pyriproxyfen in soils and plants is under the dependence of various factors [97]. Liu et al. [168] showed high differences in the toxicity of the degradation products of pyriproxyfen against adult earthworms (*Eisenia foetida*). It would be necessary to perform the same kind of work on all the different bee species because a priori there is nothing to suggest that the results obtained on the honey bee for the different metabolites could be extrapolated to the other bee species.

Devillers et al. [121] and Devillers and Devillers [141] demonstrated the utility of a population dynamics model for simulating the long-term effects of sublethal concentrations of juvenile hormone mimics on the different inhabitants of a hive. They also revealed that it was possible to study simultaneously the effects of stressors observed at different organization levels within the hive. Whereas the number of ecotoxicological studies on the non-*Apis* bees is rather limited, on the contrary, the ecology of some representative species is well documented. As a result, there exist population dynamics models that could be used [169,170,171,172], directly or after some refinements, for estimating the long-term effects of juvenile hormone mimics on the non-*Apis* bees with realistic field scenarios.

## 5. Conclusions and Recommendations

Whereas the ecotoxicity of pyriproxyfen on the honey bee (*A. mellifera*) is well documented, there is a crucial need for laboratory and field studies for evaluating the adverse effects of this insecticide on the bumble bees, the solitary bees, and the stingless bees. These studies must focus in priority on sublethal toxicity effects and have to be made on at least one representative species of each category of non-*Apis* bees.

In addition, although pollen and nectar are the most common routes of exposure of bees to insecticides, there are also differences depending on their life history traits. Taking these differences into account is crucial for chemicals targeting the development of the insects such as pyriproxyfen. There is a need to identify all the direct and indirect routes of exposure of the bumble bees, solitary bees, and stingless bees to pyriproxyfen.

The potential adverse effects of pyriproxyfen on the reproductive success of the *Apis* and non-*Apis* bees are unknown and have to be evaluated. The toxicity of its metabolites is also unknown and needs to be assessed due to the fate of pyriproxyfen in terrestrial ecosystems.
